# Measuring National Immunization System Performance: A Systematic Assessment of Available Resources

**DOI:** 10.9745/GHSP-D-22-00555

**Published:** 2023-06-21

**Authors:** Cyra Patel, Nicole Rendell, Ginny M. Sargent, Akeem Ali, Christopher Morgan, Rebecca Fields, Meru Sheel

**Affiliations:** a National Centre for Epidemiology and Population Health, Australian National University, Acton, Australia.; b World Health Organization, Seoul, Republic of Korea.; c Jhpiego, Baltimore, MD, USA.; d School of Population and Global Health, University of Melbourne, Melbourne, Australia.; e Burnet Institute, Melbourne, Australia.; f JSI Research & Training Institute, Inc., Arlington, VA, USA.; g Sydney School of Public Health, Faculty of Medicine and Health, The University of Sydney, Camperdown, Australia.

## Abstract

**Background:**

Vaccination coverage is widely used to assess immunization performance but, on its own, provides insufficient information to drive improvements. Assessing the performance of underlying components of immunization systems is less clear, with several monitoring and evaluation (M&E) resources available for use in different operational settings and for different purposes. We studied these resources to understand how immunization system performance is measured.

**Methods:**

We reviewed peer-reviewed and gray literature published since 2000 to identify M&E resources that include national-level indicators measuring the performance of immunization systems or their components (governance, financing, regulation, information systems, vaccine logistics, workforce, service delivery, and demand generation). We summarize indicators by the system components or outcomes measured and describe findings narratively.

**Results:**

We identified 20 resources to monitor immunization program objectives and guide national strategic decision-making, encompassing 631 distinct indicators. Indicators for immunization program outcomes comprised the majority (124/631 [19.7%]), largely vaccination coverage (110/124 [88.7%]). Almost all resources (19/20 [95%]) included indicators for vaccine logistics (83/631 [13.2%]), and those for regulation (19/631 [3.0%]) and demand generation (28/631 [4.4%]) were least common. There was heterogeneity in how information systems (92/563 [14.6%]) and workforce (47/631 [7.4%]) were assessed across resources. Indicators for vaccination coverage in adults, data use in decision-making, equity and diversity, effectiveness of safety surveillance, and availability of a public health workforce were notably lacking.

**Conclusions:**

Between the resources identified in this review, we identified considerable variability and gaps in indicators assessing the performance of some immunization system components. Given the multitude of indicators, policymakers may be better served by tailoring evaluation resources to their specific context to gain useful insight into health system performance and improve data use in decision-making for immunization programs.

## Abstract (precis)

This review of monitoring and evaluation resources identified multiple performance indicators that can inform country-specific approaches to evaluating immunization systems.

## Teaser

**Key Findings:**

- We reviewed monitoring and evaluation (M&E) resources to understand how national immunization system performance has been measured, identifying 631 distinct M&E indicators that reflect the increasing complexity of immunization programs and the changing policies, priorities, and reporting requirements of immunization actors at the global level.
- This study highlights the substantial workload associated with the large burden of data collection and routine reporting for immunization alone, especially in resource-constrained settings.
- Despite the large number of indicators identified, gaps remain in measuring key areas of immunization, such as adult immunization, data use in decision-making, and equity and diversity, where efforts are needed to develop and validate indicators.

**Key Implications:**

- This review can inform country-specific approaches to measuring system performance moving forward, particularly as countries seek to strengthen immunization programs by leveraging investments made during the COVID-19 response.
- Public health practitioners can streamline how immunization is measured by selecting a core set of indicators that align with the priorities, values, and challenges within their specific setting.

## INTRODUCTION

Successful immunization programs are strongly associated with lower childhood morbidity and mortality and are an important marker of health systems performance.^1^^–^^3^ As an essential health service, immunization performance is routinely measured by vaccination coverage.^1^ Despite the increased breadth of protection through new vaccine introduction, coverage rates of vaccines given in infancy have stagnated in the last decade and remain lower than the World Health Organization’s goal of 90%.^4^^,^^5^ Immunization successes gained globally between 2010 and 2019 were adversely impacted by the COVID-19 pandemic.^6^^,^^7^ In 2020, coverage rates dropped to 83% for the third dose of diphtheria-tetanus-pertussis vaccine (DTP3) and 84% for the first dose of measles-containing vaccines^8^ and then even further in 2021 (DTP3: 81%; first dose of measles-containing vaccine: 81%).^9^ The number of zero-dose children, defined as infants who did not receive any doses of the DTP vaccine, was reported as high as 18.2 million in 2021.^10^

Measuring vaccine and zero-dose coverage alone is insufficient to drive improvements in immunization. An immunization system contains health system components, including all the organizations, institutions, resources, processes, and activities involved in the delivery of vaccines.^11^ Vaccines of assured quality must be available at the point of service, trained health workers must administer vaccines, and the community must demand vaccines and be able to access them.^1^ Program delivery is underpinned by dedicated financing and governance structures and surveillance mechanisms.^1^ High-quality and timely data inform where systematic weaknesses exist and how resources should be invested.^1^^,^^12^ System components need to work together effectively to achieve high vaccine coverage.^1^^,^^13^ In rural Madagascar, for example, interventions to improve infrastructure, staff training and availability, and procurement systems, underpinned by data from a new health information system platform, increased full vaccination coverage among children aged 12–23 months from 34.6% in 2014 to 63.6% in 2018.^14^^,^^15^ Initiatives like these demonstrate the importance of assessing immunization system components to target and prioritize strategies to improve overall performance.

Unlike immunization coverage, there is no clear consensus on how to measure the performance of immunization system components. Several monitoring and evaluation (M&E) resources exist to measure the performance of immunization systems or programs and are used for different purposes.^16^^–^^18^ In planning for the integration of COVID-19 vaccination programs into immunization systems, there is a strong focus on integrating not just health services but also health governance functions and on improving the availability and reach of health services across the life course.^19^ There are opportunities to capitalize on the investments made during the COVID-19 pandemic, such as through leveraging technological advancements, enhancing disease surveillance and monitoring of adverse events following immunization, enumerating and upskilling workforce, and strengthening supply chains for vaccines and other medications.^19^ Examining the current performance of immunization systems can help countries understand how best to achieve integration and leverage the investments made for the COVID-19 response. This creates a timely opportunity for the review of existing M&E resources. In this study, we examine how immunization system performance has been measured in the past and where gaps exist in measuring the performance of specific components of the system.

Examining the current performance of immunization systems can help countries understand how to achieve integration and leverage COVID-19 response investments.

## METHODS

We reviewed existing M&E resources used for immunization systems globally to understand how immunization system performance is measured. We were guided by the Preferred Reporting Items for Systematic Reviews and Meta-Analyses guidelines.^20^

### Conceptualization of an Immunization System

We applied the World Health Organization’s (WHO) health system framework to categorize indicators by the components of immunization systems that they intend to measure. The framework describes health systems according to 6 building blocks^21^ and is frequently used to examine system-wide impacts of initiatives like the introduction of a new vaccine.^22^^,^^23^ The 6 building blocks include workforce, information systems, supply chains, financing, governance, and service provision. Additionally, we included safety and regulation of vaccines and demand generation in our framework because the published literature recognizes these components as critical.^24^^,^^25^

We defined the outcomes of immunization systems as the immediate and short-term effects achieved through the collective and synchronized functioning of immunization system components, namely, to vaccinate populations against a broad range of vaccine-preventable diseases. Impacts of immunization systems were defined as health effects and changes in disease burden associated with vaccine-preventable diseases and overall effects on the health of populations. This conceptualization was the basis for the immunization system framework developed as part of this study (Supplement 1).

### Search Strategy

We searched for M&E resources on immunization systems in the peer-reviewed (OVID MEDLINE) and gray literature (Google Scholar) using search terms related to immunization systems, evaluation, indicators, measurement, and surveillance. The detailed search strategy is available in Supplement 2. Articles were also identified by snowballing through references of included publications. Searches of the gray literature were limited to M&E resources published by global partner agencies. We excluded international donors and funders not directly involved in implementing programs due to the differing priorities and motivations of these agencies and potential/perceived conflict of interest. Searches were conducted between March (MEDLINE) and May (Google Scholar) 2022.

### Inclusion and Exclusion Criteria

#### M&E Resources of Immunization Systems

We included contemporary M&E resources published in 2000 or later that examined system-wide immunization performance. Our selection process is illustrated in the Figure, and the inclusion and exclusion criteria are summarized in the Box. We considered a resource eligible if it (1) incorporated national or country-level indicators that measured the overall performance of the immunization system and at least 1 of its components or (2) examined the performance of multiple components of the system. Resources that included only outcome (e.g., vaccination coverage) or impact-level indicators (e.g., disease incidence or mortality) or that included indicators only at the global, regional, or subnational levels were excluded. We excluded resources that focused on a single aspect of the immunization system (i.e., did not examine overall performance or other components of the system) and studies that evaluated a specific intervention or targeted initiative that did not examine system-wide performance (e.g., implementation of a new logistics management system that only evaluated the direct benefits of supply chain management improvement, without consideration of service provision or coverage of vaccines). Similarly, we excluded M&E resources that were focused on evaluating a disease-specific program and did not include indicators examining the impact of the program on the immunization system. Titles and abstracts were screened by 1 author (CP) using Rayyan QCRI.^26^ Ambiguous resources were discussed with MS. Two authors (CM and RF) who are content area experts also reviewed the list of resources identified to ensure it was comprehensive.

**FIGURE:**
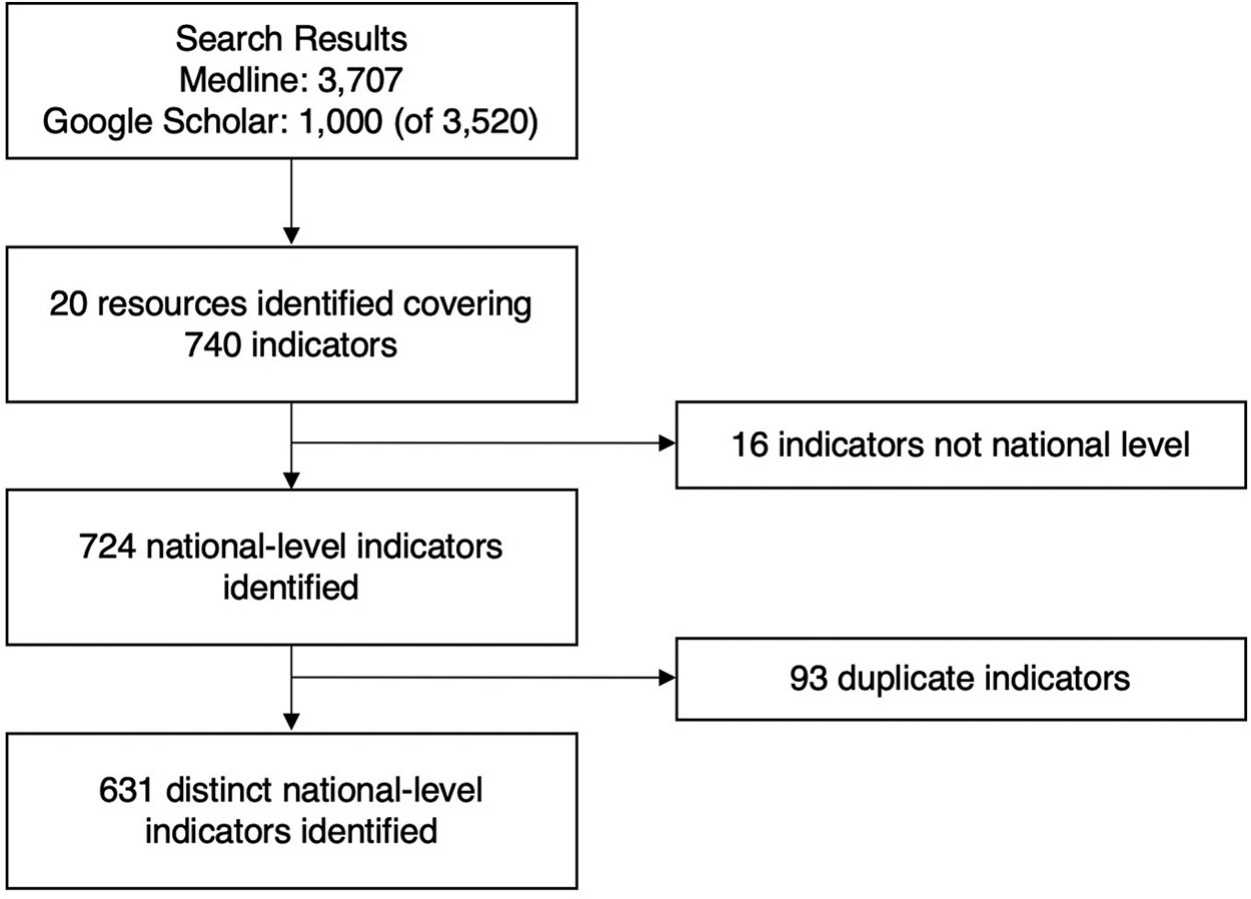
Search Results and Included Resources on Immunization Monitoring and Evaluation

BOX Inclusion and Exclusion Criteria for Review of Monitoring and Evaluation Resources Measuring National Immunization System Performance

**Inclusion criteria**

- Examines system-wide performance of immunization system or the system-wide impact of a disease-specific immunization program or initiative
- Includes performance indicators on overall system performance and at least 1 of the system components or examines the performance of multiple components of the system
- Incorporates national-level indicators
- Published in 2000 or later

**Exclusion criteria**

- Included only outcome (e.g., vaccination coverage) or impact-level indicators (e.g., disease incidence)
- Did not include national-level indicators (e.g., resources measuring performance at the subnational level only)
- Focused on a single component of the immunization system without examining system-wide performance
- Evaluated a disease-specific immunization program, intervention, or targeted initiative without examining its impact on system-wide performance

#### Immunization System Indicators

Following extraction of all indicators from the included M&E resources, we included all indicators where the value was numeric or categorical (e.g., yes/no or high/medium/low responses). We included indicators measuring performance at a global level that could be modified to the national level, as data for these indicators are typically collected nationally and then aggregated to obtain a global estimate (e.g., “proportion of countries with DTP vaccine coverage ≥80%” was extracted as “national DTP vaccine coverage is ≥80%”). We excluded indicators that were not applicable at the national level related to goals requiring global effort and coordination, such as development of new vaccine platforms. In our examination of distinct indicators, we removed indicators that were duplicated across resources.

### Data Extraction and Analysis

We tabulated data on the M&E resources, including the tool’s author, year of publication, purpose, context or location where it was used, and the number of indicators in each tool. We used methods previously used to summarize and compare performance indicators, particularly those used to analyze indicators for health system performance.^27^^,^^28^ We extracted and classified indicators into 10 broad domains aligning with system impacts, system outcomes, and the 8 system components. We further categorized the indicators within each domain into measurement areas to better understand how performance within each domain is assessed (see Supplement 1 for definitions). A formal quality assessment of resources and indicators was not done.

Data were extracted using Microsoft Excel and imported into NVivo 12 for coding. Two authors (CP and NR) independently coded the indicators. Discrepancies were resolved through discussion and consensus. We categorized each indicator into a single domain and measurement area. Where indicators could be categorized under 2 or more system domains, we used contextual information from the source document to understand which domain the indicator was intended to measure. For example, “the proportion of the immunization budget dedicated to advocacy activities” was categorized under demand generation rather than financing because it was used in the source document to measure commitment to engaging with communities to build demand for vaccines.

We used summary statistics to calculate (1) the number of M&E resources, including indicators for each domain and measurement area, and (2) the number of indicators in each domain and measurement area. We summarize our findings narratively.

## RESULTS

After screening 3,707 titles in the peer-reviewed literature and 1,000 in the gray literature, we identified 20 M&E resources that met the inclusion criteria (Table 1); 13 from the gray literature (of which 1 was an unpublished draft document)^13^^,^^17^^,^^18^^,^^29^^–^^38^ and 7 from the peer-reviewed literature.^1^^,^^39^^–^^44^ M&E resources from the gray literature were published by global partner agencies, including the World Health Organization (WHO) (n=8, 1 in collaboration with UNICEF);^13^^,^^29^^–^^31^^,^^33^^,^^35^^,^^37^^,^^38^ Gavi, the Vaccine Alliance (n=2);^17^^,^^36^ and the U.S. Agency for International Development (n=2).^18^^,^^32^ The remaining resource from the gray literature was developed by an independent group.^34^

**TABLE 1.** Summary of Included Publications Reporting Immunization Program Monitoring and Evaluation Resources

**Authors, Year** ^a^	**Name of Tool**	**Publication Type and Purpose**	**Location/Context for Use**	**Indicators, No.**
Sodha and Dietz, 2015^1^	Indicators that can be used to monitor immunization performance	Peer-reviewed literature; provides examples of multiple indicators necessary to monitor various components of immunization programs and to assess overall program performance.	All countries, but particularly LMICs	21
WHO, 2020^13^	Immunization Agenda 2030 Monitoring and Evaluation Framework	Gray literature; to measure progress toward the goals and objectives of IA2030 strategy and enable use of data for action to continuously improve immunization programs at the national, regional, and global levels.	All countries	112
Gavi, 2018^17^	Gavi 2016–2020 Strategy Indicators	Gray literature; to measure progress toward achieving 4 goals of Gavi’s 5-year strategy for 2016–2020.	Gavi-supported countries	27
USAID, 2017^18^	USAID MCSP indicators that describe the strength of the routine immunization system	Gray literature; to describe and measure functioning of immunization system in real time to provide managers with information on strengths and gaps in immunization system and to inform actions for improving vaccination coverage and help explain reasons for low coverage.	LMICs (with a focus on African countries)	10
WHO and UNICEF, 2019^29^^,^^b^	WHO–UNICEF Joint Reporting Form on Immunization	Gray literature; to collect countries’ annual immunization data in standardized format to help identify trends and gaps at the country, regional, and global levels.	All countries	169
WHO, 2018^30^	Reaching Every District Monitoring Tool	Gray literature; to provide a guide for monitoring immunization programs for district health management teams and health facilities.	LMICs, particularly African countries	12
WHO, 2017^31^	A Guide for Conducting an Expanded Programme on Immunization (EPI) Review	Gray literature; to comprehensively assess strengths and weaknesses of an immunization program at national, subnational, and service-delivery levels to provide evidence for program’s strategic directions and priority activities.	All countries	85
USAID, 2016^32^	USAID Monitoring and Evaluation of the Reaching Every Child-Quality Improvement (REC-QI) approach	Gray literature; to assess ability of the REC-QI approach to improve functionality, efficiency, and sustainability of routine immunization system and to assess how key components of REC-QI model contribute to strong routine immunization system.	LMICs (with a focus on African countries)	19
WHO, 2010^33^	New Vaccine Post-Introduction Evaluation (PIE) Tool	Gray literature; to evaluate overall impact of introduction of a new vaccine on country’s national immunization program.	All countries	16
Griffith et al., 2010^34^	Toolkit for assessing the impact of measles eradication activities on immunization services and health systems	Gray literature; to assess impact of measles elimination activities on goals related to elimination of measles and impact on routine immunization services and the health system.	Global with a focus on LMICs, especially countries with measles elimination programs	11
WHO, 2002^35^	WHO Common Assessment Tool for Immunization Services	Gray literature; to assess immunization services in the wider context of the health system.	All countries	55
Gavi, 2002^36^	Gavi and WHO Monitoring National Immunization Systems Using Core Indicators	Gray literature; to monitor progress toward immunization system targets, and to identify and analyze problems that can guide management decisions.	Gavi-supported countries	23
WHO, 2001^37^	Checklist and indicators for optimizing the impact of polio activities on EPI (draft)	Gray literature; to help national decision-makers and program managers to maximize positive impacts of polio eradication on routine immunization services.	LMICs, especially those with substantial polio eradication activities	10
WHO, (unknown)^38^	WHO Indicators for Monitoring District and National Performance	Gray literature; to monitor all components of immunization systems and draw attention to low-performing areas that need additional support to improve access and increase district-level vaccine coverage (monitoring both district and national levels).	All countries, with a focus on LMICs	40
Cernuschi et al., 2018^39^	Gavi indicators for sustainable immunization systems	Peer-reviewed literature; to analyze the sustainability of immunization programs in Gavi transitioning countries and identify potential sustainability issues, particularly in 4 programmatic areas: (1) decision-making, (2) political commitment and financial sustainability, (3) demand for and equitable delivery of vaccines, and (4) access to timely and affordable supply.	Gavi-supported countries	14
National Vaccine Advisory Committee, 2017^40^	Proposed indicators to advance vaccine and immunization efforts in the United States	Peer-reviewed literature; to measure success and monitor progress on established target goals corresponding to 5 opportunity areas for advancing U.S. vaccine and immunization efforts: (1) strengthen health information and surveillance systems, (2) strengthen confidence in vaccines and improve coverage across life span, (3) eliminate financial and systems barriers to vaccination, (4) strengthen the science base for developing and licensing vaccines, (5) facilitate vaccine development.	United States	32
Poy et al., 2017^41^	Indicators for Immunization Systems Management Group Routine Immunization Dashboard	Peer-reviewed literature; to monitor progress in routine immunization through a dashboard using agreed standard indicators that reflect steps in the pathway of routine immunization strengthening with a focus on polio high-risk districts.	LMICs, especially those with polio eradication programs	15
Tegegne et al., 2016^42^	Accountability framework for the Nigeria polio program	Peer-reviewed literature; to systematically monitor and evaluate the impact of the polio eradication initiative in Nigeria using indicators that cut across different program areas and to measure program and staff performance.	Nigeria	21
Shuaib et al., 2014^43^	Accountability Framework for Routine Immunization, Nigeria	Peer-reviewed literature; to monitor routine immunization administration and vaccine management and to ensure that government officials could access high-quality routine immunization data to monitor performance and improve routine immunization coverage.	Nigeria	21
WHO, 2013^44^	Global Vaccine Action Plan: Monitoring and Evaluation/Accountability Framework	Peer-reviewed literature; to monitor progress of immunization programs against Global Vaccine Action Plan goals, specifically monitoring results.	All countries	27

Abbreviations: EPI, Expanded Programme on Immunization; IA2030, Immunization Agenda 2030; JRF, Joint Reporting Form; LMIC, low- and middle-income country; MCSP, Maternal and Child Survival Program; SIA, supplementary immunization activity; WHO, World Health Organization.

a Where information was available, the year of publication denotes the year that the indicator tool was published and available for use and is not necessarily the date of publication of the article/report.

b The JRF is revised on a regular basis; this study includes indicators included in the tool in 2019.

Table 1 describes the identified M&E resources across key categories, including purpose of the resource, context where the resource is intended to be used, and the number of indicators. All resources were designed to monitor progress toward immunization program objectives and collate data to guide national-level strategic and programmatic decision-making to achieve goals. Three resources assessed the extent to which specific initiatives (namely, measles and rubella elimination programs and polio eradication programs) improved routine immunization.^34^^,^^37^^,^^42^ Three resources examined the extent to which the objectives of global development organizations were achieved.^13^^,^^17^^,^^44^ One resource (WHO’s *New Vaccine Post-Introduction Evaluation Tool*) evaluated the introduction of new vaccines into national immunization programs.^33^ One resource established for use in the United States^40^ included indicators that could be applied in other countries but is likely to be of limited use in low-and-middle-income countries (LMICs) due to the differing priorities and degree of maturity of immunization systems compared with high-income countries.

The 20 included resources captured a total of 740 indicators, of which 631 distinct indicators (85%) were retained following exclusion of ineligible and duplicate indicators (Figure and Box). Table 2 presents the number of resources and corresponding distinct indicators covering each domain. Of the 631 distinct indicators, 47 (7.4%) measured system impacts, 124 (19.7%) measured system outcomes, and 460 (72.9%) measured the performance of immunization system components. Three resources—the Immunization Agenda 2030 (IA2030) Monitoring and Evaluation Framework,^13^ the WHO-UNICEF Joint Reporting Form,^29^ and the guide for conducting an Expanded Programme on Immunization review^31^—included indicators that covered all 10 domains. Indicators from these 3 resources comprised almost half of all 740 indicators identified (112, 169, and 85, respectively, a total of 366/740 [49.5%]). A summary of which resources included indicators that measured performance in each domain is in Supplement 3.

**TABLE 2.** Number and Proportion of Included Monitoring and Evaluation Resources and Distinct Indicators Measuring Performance in 10 Indicator Domains

**Indicator Domain**	**Resources, No. (%)** **(N=20)**	**Indicators, No. (%)** **(N=631)**
Impacts of immunization system	9 (45.0)	47 (7.4)
Outcomes of immunization system	17 (85.0)	124 (19.7)
Performance of immunization system components		
Demand generation	14 (70.0)	28 (4.4)
Financing	15 (75.0)	44 (7.0)
Governance, program planning, and management	18 (90.0)	84 (13.3)
Information systems	18 (90.0)	92 (14.6)
Regulation and pharmacovigilance	8 (40.0)	19 (3.0)
Service provision	16 (80.0)	63 (10.0)
Vaccine logistics, products, and supplies	19 (95.0)	83 (13.2)
Workforce	18 (90.0)	47 (7.4)

### Performance Indicators for Immunization System Impacts and Outcomes

Table 3 summarizes the resources and indicators measuring the outcomes (n=8) and impacts of immunization systems (n=16). Nine resources (45.0%) included indicators measuring system impact, with the majority (39/47 [83.0%]) of indicators measuring incidence, mortality, or disability-adjusted life years attributable to specific vaccine-preventable diseases. In our review, indicators for immunization system outcomes, found in 17 (85.0%) resources, comprised 19.7% of all indicators (124/631). Of the 124 indicators, 110 (88.7%) related to vaccination coverage, specifically coverage of 1 or more specific vaccines in the target population (84/110), equity of coverage (17/110), or dropout rates (9/110). Indicators for childhood vaccination were most common, with 24 of 67 indicators on childhood vaccination coverage specifically examining coverage of DTP-containing vaccines. Indicators for the proportion of children fully vaccinated according to specific schedules or the proportion of children who have not received any vaccine (i.e., “zero-dose coverage”) occurred in resources published since 2015. Only 17 of the 84 indicators on vaccination coverage pertained to vaccine coverage for adolescents or adults. Four resources included 14 indicators assessing success in introducing new vaccines or sustaining the use of a recently introduced vaccine in national immunization programs. Eight of the 14 indicators (57.1%) were included in the IA2030 M&E framework.

**TABLE 3.** Summary of Included Monitoring and Evaluation Resources and Indicators for Measuring Immunization System Impacts and Outcomes

**Measurement Area**	**Resources, No. (%)** ^a^	**Indicators, No. (%)** ^b^	**Indicator Description**
Impacts			
Any	9 (45.0)	47 (100.0)	Disease burden indicators examined disease incidence, mortality, or DALYs attributable to 16 VPDs. Indicators examining elimination or eradication targets pertained to measles, rubella, and neonatal tetanus elimination and polio eradication. The 2 summary metrics of disease burden pertained to mortality rates for children aged younger than 5 years.
Disease burden due to VPDs	6 (30.0)	39 (83.0)	
Achievement of elimination or eradication target	4 (20.0)	6 (12.8)	
Occurrence of outbreaks due to VPDs	1 (5.0)	1 (2.1)	
Summary metrics of disease burden	2 (10.0)	1 (2.1)	
Outcomes			
Any	17 (85.0)	124 (100.0)	Among indicators for vaccination coverage: 74 examined coverage of specific vaccines. 24 were specifically for DTP-containing vaccines(24/74 [32.4%]); 4 were for MCV coverage (4/74 [5.4%]). 17 examined coverage for vaccines across the life span (11 for influenza vaccine, 3 for HPV vaccine, 2 for tetanus toxoid in pregnant women, 1 for herpes zoster vaccine); 14/17 were identified in the WHO-UNICEF JRF tool. 7 examined the proportion of target population “fully vaccinated.” 3 examined zero-dose vaccine coverage.5/9 distinct vaccine dropout indicators examined DTP-containing vaccine dropout.12/17 indicators on vaccine equity examined variations invaccine coverage across districts or other geographicalregions; the remainder examined variations in coverage bysociodemographic factors (wealth, education, ethnicity);none examined variation by gender.
Vaccination coverage	17 (85.0)	84 (67.7)	
Dropout of vaccination coverage	12 (60.0)	9 (7.3)	
Equity of vaccination coverage	8 (40.0)	17 (13.7)	
New vaccine introduction	4 (20.0)	14 (11.3)	

Abbreviations: DALYs, disability-adjusted life years; DTP, diphtheria-tetanus-pertussis; HPV, human papillomavirus; JRF, Joint Reporting Form; MCV, measles-containing vaccine; VPD, vaccine-preventable disease; WHO, World Health Organization.

a Denominator is the 20 resources identified in this review.

b Denominator is the number of indicators within each indicator domain.

### Performance Indicators for Immunization System Components

The number of resources and indicators measuring the performance of immunization system components is summarized in Table 4. Almost all resources covered metrics for vaccine logistics (19/20 [95.0%]). Indicators for workforce, information systems, and governance were each included in 18 resources (90.0%). Indicators for information systems (92/631 [14.6%]), governance (84/631 [13.3%]), and vaccine logistics (83/631 [13.2%]) were the most frequent.

**TABLE 4.** Summary of Included Monitoring and Evaluation Resources and Indicators Measuring the Performance of Immunization System Components

**Indicator Domain and Measurement Area**	**Resources, No. (%)** ^a^	**Indicators, No. (%)** ^b^	**Indicator Description**
Demand generation			
Any	14 (70.0)	28 (100.0)	Indicators for community engagement examined how frequently activities to engage communities occurred (e.g., the number of meetings occurring in communities to discuss immunization); planning and financing these activities, including documentation of planned strategies; and inclusion of community representatives in program planning. Indicators assessing vaccine demand, knowledge and confidence included those assessing if the public were demanding vaccines, support for immunization by community leaders, whether strategies were being implemented to improve communication and demand for vaccines, and if systems were in place to measure vaccine confidence at a national or subnational level. Examples of indicators include “percentage of un- and under-vaccinated in whom lack of confidence was a factor that influenced their decision” or if staff at health facilities received training on communication.
Community engagement	12 (60.0)	16 (57.1)	
Vaccine demand, knowledge, and confidence	7 (35.0)	12 (42.9)	
Financing			
Any	15 (75.0)	44 (100.0)	Under financial planning, indicators assessed if actual expenses were consistent with budgets, accounting practices, if funds were disbursed in a timely manner, and if the allocated funds were adequate to meet program objectives; 1 indicator examined if activities were canceled due to lack of funds. Indicators for government spending assessed the dollar value of government spending on immunization programs and the proportional contribution of government spending relative to total spending on immunization. Costs of vaccines and programs examined trends in costs over time.
Government spending on immunization	10 (50.0)	18 (40.9)	
Total expenditure (all sources)	3 (15.0)	4 (9.1)	
Financial planning	10 (50.0)	20 (45.5)	
Costs of vaccines and programs	2 (10.0)	2 (4.5)	
Governance, program planning, and management			
Any	18 (90.0)	84 (100.0)	The majority of indicators (38/84) examined if specific policies, processes, and plans were in place (but not necessarily whether they were implemented or enforced). Examples include if annual or multiyear national plans for immunization were available, if microplanning was conducted at subnational levels, if plans included strategies for hard-to-reach populations, and if specific national policies (e.g., such as waste management or injection safety) were available. Indicators pertaining to program management included 3 examining the existence of a national technical advisory group on immunization and an additional 9 indicators examining specific characteristics of this group. Program coordination indicators assessed if coordination and communication across different levels of the health system occurred (e.g., multisector coordination mechanisms functional at all levels and staff at all levels receive timely information on new policies and guidelines.) Indicators for processes for monitoring and evaluation examined if measures for ensuring accountability in immunization programs were in place (e.g., through an evaluation framework or other means to monitor performance, provision of feedback to subnational levels, and engagement of various stakeholders in monitoring immunization programs).
Existence of policies, processes, and plans	13 (65.0)	38 (45.2)	
Program management	9 (45.0)	20 (23.8)	
Program coordination	6 (30.0)	12 (14.3)	
Plan or process for monitoring and evaluation	6 (30.0)	14 (16.7)	
Information systems			
Any	18 (90.0)	92 (100.0)	Indicators for data quality included those measuring completeness and timeliness of reporting to higher levels of the health system, the accuracy of data (e.g., through coverage rates greater than 100% or negative values for coverage dropout rates or if denominator data are accurate) and assessing if data on certain variables is collected (e.g., age data on cases of VPDs). Indicators for disease surveillance examined the ability to detect specific diseases of interest such as polio, measles, and rubella, including laboratory capacity and capability to test for VPDs. Indicators on processes and systems examined whether certain systems existed to collect and report data on immunizations and how widespread access to them was (e.g., the proportion of the population with access to immunization records). This included 7 indicators on the use of technology such as digital tools (e.g., electronic stock management system) or electronic means of entering and transmitting data. Indicators on data use examined whether data was used to inform plans for delivering routine immunization services and in outbreak response campaigns.
Data quality	15 (75.0)	37 (40.2)	
Use of data	4 (20.0)	5 (5.4)	
Immunization data systems and processes	6 (30.0)	26 (28.3)	
VPD surveillance	8 (40.0)	24 (26.1)	
Regulation and pharmacovigilance			
Any	8 (40.0)	19 (100.0)	Indicators for safety surveillance examined whether systems to detect adverse events related to immunization existed and the number and rate of adverse event reports. Indicators assessing regulatory policies and processes largely examined whether certain policies and procedures to ensure the safety of vaccine products (e.g., the proportion of vaccines procured of assured quality or existence of guidance on waste management) and vaccine administration (e.g., completion of a standardized injection safety assessment) were in place.
Safety surveillance	6 (30.0)	11 (57.9)	
Regulatory policies and processes	6 (30.0)	8 (42.1)	
Service provision			
Any	16 (80.0)	63 (100.0)	Indicators on the provision of immunization services examined the number of fixed and/or outreach immunization sessions conducted, the number of sessions conducted relative to those planned, and the number of doses of specific vaccines administered. Two tools (the IA2030 M&E framework and guide to conducting an EPI review) included indicators examining plans for reaching zero-dose and under-vaccinated populations but did not assess if related goals were achieved. Indicators measuring integration largely examined the provision of immunization alongside other health services (11/19), usually primary health care services like vitamin A and antenatal care. Some examined provision of immunization and other services like tertiary services and in settings like schools and pharmacies.
Provision of immunization services	14 (70.0)	39 (61.9)	
Activities to reach disadvantaged or under-immunized populations	2 (10.0)	5 (7.9)	
Integration of immunization with other healthservices	7 (35.0)	19 (30.2)	
Vaccine logistics, products, and supplies			
Any	19 (95.0)	83 (100.0)	Indicators on availability of vaccines and supplies pertained to the availability of products at the point of service (i.e., occurrence of stock-outs of vaccines and supplies). Indicators examining effective management of vaccines related to cold chain management (e.g., proportion of facilities with functional refrigerators or with temperature monitored), supply chain management (e.g., use of vaccine forecasting processes and kilometers per vehicle to transport vaccines) and management of waste (e.g., wastage rates of closed vials and availability of adequate infrastructure and supplies for waste management). Indicators related to innovations in vaccine products examined the use of innovative products (e.g., newly recommended vaccines or new technologies to deliver vaccines) and capacity to conduct vaccine research, particularly clinical trials.
Availability of vaccines and supplies	18 (90.0)	40 (48.2)	
Effective management of vaccines	13 (65.0)	37 (44.6)	
Use of innovation	1 (5.0)	6 (7.2)	
Workforce			
Any	18 (90.0)	47 (100.0)	Indicators for training and supervision of health workers examined whether supervisory visits and opportunities for training occurred (e.g., number or proportion of health facilities reached with supportive supervision) and if the training and feedback provided was adequate (e.g., proportion of staff satisfied with training). Indicators on availability of health workers assessed the size of the workforce, focusing on the clinical workforce (e.g., number of health workers per 10,000 population) and gaps in workforce (e.g., the ratio of unfilled to total number of posts). Three indicators examined whether there were adequate workforce for nonclinical immunization functions, (i.e., data management, human resources, and supply chain management). Indicators on health worker competency examined the proportion of staff who were able to conduct certain tasks correctly (e.g., providing correct case definition for a disease).
Training and supervision of health workers	14 (70.0)	20 (42.6)	
Availability or quantity of health workers	9 (45.0)	15 (31.9)	
Health worker competence	6 (30.0)	8 (17.0)	
Working conditions	2 (10.0)	4 (8.5)	

Abbreviations: EPI, Expanded Programme on Immunization; IA2030, Immunization Agenda 2030; M&E, monitoring and evaluation; VPD, vaccine preventable disease.

a Denominator is the 20 resources identified in this review.

b Denominator is the number of indicators within each indicator domain.

Among indicators for vaccine logistics and supplies, almost half measured availability of supplies at point of service through frequency and duration of stockouts (40/83 [48.2%]). Indicators assessing the performance of cold chain and supply chain management and functionality were also common (37/77 [44.6%]). Among indicators for service provision, those examining the provision of services through the number of vaccination sessions held or the number of doses given were common across resources (39/63 [61.9%]). Nineteen indicators examined integration (i.e., the co-delivery of immunization with other health services), with all but 1 of them from resources published in 2017 onwards. Nearly half of the 84 indicators for governance, planning, and program management (38/84 [45.2%]) assessed whether specific policies, protocols, or processes were in place. Twelve of 20 indicators assessing program management pertained to the existence or features of national immunization technical advisory groups.

Indicators for information systems and workforce varied to a greater extent than the other domains. Under information systems, most indicators measured data quality (37/92 [40.2%]) and the existence of processes to collect immunization (26/92 [28.3%]) and disease surveillance data (24/92 [26.1%]). Although indicators examined whether specific processes, systems, or resources were available in the country, none examined their functionality, use, or acceptance by workers, and only 1 examined whether disease surveillance and immunization data systems were interoperable. Five indicators (5.4%) measured data use. Most indicators examining the performance of workforce pertained to the availability of health workers for immunization (20/47 [42.6%]) or the frequency and quality of supervision (15/47 [31.8%]). Those on health worker availability largely measured the density of the clinical workforce; 3 assessed the availability of workforce supporting nonclinical functions in the immunization system (specifically data management, human resources, and supply chain management), but none assessed the size or capacity of the public health workforce.

Indicators measuring the performance of regulation and pharmacovigilance and demand generation were the least frequent (19/631 [3.0%] and 28/631 [4.4%], respectively). Indicators for pharmacovigilance and regulation appeared in the fewest number of resources (8/20 [40.0%]). Indicators for safety surveillance largely included those for rates of adverse event reporting and whether a system for safety surveillance exists. Under demand generation, most indicators identified focused on the occurrence of community engagement sessions (16/28 [57.1%]). Indicators for assessing the level of demand for vaccines varied substantially but mostly examined if strategies were being implemented to improve communication and demand for vaccines and if systems were in place to measure vaccine confidence.

## DISCUSSION

Our study found a multitude of indicators examining the performance of immunization systems and their underlying components, with 631 distinct indicators across 20 M&E resources. We identified variations in how domains were measured, including those where there was some consistency across resources, like those for coverage, service provision, and vaccine supplies and logistics. Although some differences may be due to the differing purposes of the resources or operational contexts in which they are used, the differences and multiplicity of resources reflect the increasing complexity of immunization programs and the changing policies, priorities, and reporting requirements of immunization actors at the global level. Recently, global focus has shifted from achieving high coverage of individual vaccines through disease-specific initiatives to achieving universal health coverage through system-wide strengthening approaches.^5^^,^^45^ Zero-dose coverage has notably emerged as a metric for childhood immunization performance, featured prominently in the IA2030 framework and Gavi’s 2021–2025 strategy.^5^^,^^13^^,^^46^

The differences and multiplicity of identified resources reflect the increasing complexity of immunization programs and global changes in policies, priorities, and reporting requirements.

Global attention is also increasing toward expanding immunization across the life span, but our review found that indicators measuring vaccination coverage in adolescence and adulthood are limited in number and scope. Fourteen of 17 indicators examining vaccination after early childhood were identified from the WHO-UNICEF Joint Reporting Form, and 11 of these pertained to influenza vaccine uptake in high-risk populations. COVID-19 vaccination is the first large-scale program targeting adults globally. Before this, 62% (120/194) of countries reported having at least 1 adult immunization program, but high- and upper-middle-income countries were almost 22 times more likely to have such a program compared with low- and lower-middle income countries.^47^ Our review did not identify any metrics examining the burden of well-established diseases affecting older adults, like influenza, pneumococcal disease, and herpes zoster. Indicators on both disease incidence and disease surveillance systems are focused on childhood diseases and disease-specific programs, such as polio eradication and measles elimination. Lack of data on the disease burden and the potential public health impact of vaccinating against these diseases in LMICs hinders decision-making related to introducing these programs.^47^^,^^48^ Enhancing surveillance for diseases affecting adults and reporting these data stratified by age and sex will require substantial investment, political will, and advocacy at global and national levels, but it is a necessary precursor to introducing adult vaccination programs in LMICs.

We found that the processes by which immunization data are collected, analyzed, reported, and accessed, and the systems used to do so, are not consistently measured across resources. Most indicators examined whether a system was in place but not its functionality, acceptability, or use. Experience with interpreting data collected and reported at a global level highlights the data quality issues that arise. For example, data on 14 of the 27 indicators proposed to measure progress against the goals of the Global Vaccine Action Plan (which preceded the IA2030 strategy) were difficult to interpret due to poor data quality and variability in reporting across countries.^49^ One indicator in the plan, “immunization coverage data assessed as high quality by WHO and UNICEF,” was abandoned due to an inability to find a suitable measure.^49^ Data on denominators based on births and deaths statistics are known to be inaccurate and incomplete in LMICs, particularly at subnational levels.^12^^,^^50^ In 2016, 76 of 96 countries reported at least 1 district with DTP3 vaccine coverage greater than 100%.^51^ Unreliable data quality means that wide variations are difficult to interpret, as it is unclear if the variations are an artifact of the data or if they are true variations.^49^ Noting the importance of robust information systems to track coverage and other immunization targets,^12^^,^^50^ newer resources are available such as the Data Quality Self-Assessment tool developed by the WHO Immunization Analysis and Insights Unit.^52^ Despite improved data quality over the last 2 decades, gains were not universal, with resource-constrained countries and those with lower immunization performance continuing to have limited to poorer quality data.^53^

Although data on numerous indicators are often collected and reported by countries, we identified few indicators in the resources included in our review that measured data-driven decision-making and program planning at the national level. Availability of data does not necessarily translate into action; mechanisms and accountability frameworks to incorporate data into decision-making are needed. Interventions to improve data use can drive improvements in data quality and increase demand for better data.^54^ A systematic review of the DHIS2 in 11 countries found that access to data increased a sense of ownership and responsibility for the quality of data, fostering a culture of data use and improvement.^55^ Although the need for including indicators on data use in M&E resources is clear, identifying user-friendly, valid indicators for data use has proven difficult, with no standard or widely accepted approach to defining or measuring data use available.^56^ Achieving consensus on what constitutes data use can help to develop performance metrics to measure data use and is an area for future research, particularly given the recent focus on improving health information systems through the implementation of digital technologies. One tool that addresses this, albeit within a specialized system component, is the Effective Vaccine Management assessment tool (excluded from our review for reasons described below), as its indicators provide the basis for recommending actions that can lead to cold chain or other supply chain improvements. Although our review focuses on the national level, data use at subnational and local health facility levels is critical to improving coverage, for example, through tracking and identifying defaulters and under-vaccinated populations, improving vaccine supply management and planning of immunization services, and tailoring strategies to build demand for vaccination.^12^ With the increasing focus on improving the availability and use of subnational-level data by organizations like Gavi,^57^ the need for performance metrics measuring data use will likely grow.

Although countries collect and report data on numerous indicators, we identified few indicators for data-driven decision-making and program planning at the national level.

We identified few indicators measuring the performance of regulation and pharmacovigilance systems for vaccines, likely because efforts to implement and strengthen national safety surveillance for vaccines are relatively more recent compared to initiatives targeting other components of the immunization system. The impetus to introduce and enhance safety surveillance systems increased for COVID-19 vaccination.^58^ The rate of adverse events following immunization (AEFI) reporting, measured as the annual AEFI reports per 100,000 surviving infants, has been proposed as a suitable indicator to monitor the performance of AEFI surveillance systems^59^ and was 1 of the more common of the 9 indicators for safety surveillance identified in this study. The number of countries meeting this reporting target has grown from 80 (41% of 194 countries) in 2000 to 109 (56%) in 2019 but varied by region.^60^ However, more granular indicators that more accurately capture the quality, functionality (e.g., ability to assess causality of AEFIs), and effectiveness of these systems are needed. We also did not identify any indicators examining the existence or functionality of no-fault vaccine injury compensation schemes, although the call to implement these, particularly in countries with more advanced safety surveillance systems, is growing.^61^

Our review found that few M&E resources measuring the performance of immunization systems include indicators that measure the confidence in vaccines or social and behavioral drivers of demand for immunization, despite growing concerns over vaccine hesitancy.^62^ Data on how countries routinely and systematically track vaccine confidence is currently lacking. Measuring vaccine demand has proved to be a challenge; the indicators identified in this review are limited in their interpretation and utility, as it is unclear how concepts such as “supports vaccination” or “lack of confidence” were defined. The lack of consistency in definitions is echoed in a review examining how outcomes in trials of childhood vaccination communication interventions are measured.^63^ Composite indicators based on qualitative data collected via the WHO-UNICEF Joint Reporting Form were the primary way to monitor vaccine demand globally^64^ but were criticized for the lack of clarity in defining what was being measured.^49^ In another review, 12 of 14 measures of confidence in childhood vaccination identified were developed and validated in high-income countries.^65^ The recently published resources for behavioral and social drivers of vaccination^66^ provide a framework to assess reasons for under-vaccination, which may address the gaps and lack of consistency in metrics for this domain and can contribute to formative work on program strengthening.

Indicators examining equitable coverage largely focused on disparities by geographical areas (such as districts), and 5 examined coverage by socioeconomic status. Our review identified some disease burden indicators disaggregated by sex but none for coverage or any that examined or collected data on the role of gender and diversity in making decisions about or implementing immunization programs. Despite known differences in immune responses to certain infections and adverse events following immunization, studies of vaccine effectiveness and safety often do not report results by gender.^67^^,^^68^ Evidence suggests that gender influences health status, access to resources and health services, decision-making autonomy, and the quality of health services.^69^ Yet, only 5 of 58 countries with COVID-19 vaccine policies in March 2021 referred to gender, and 34 of 180 countries reported sex-disaggregated data on COVID-19 vaccine coverage between April and May 2021.^70^ The absence of indicators in our study on disability, diversity, and inclusion was also a gap. Achievement of the IA2030 goals of reducing zero-dose children and improving equity of coverage across the life span will require identifying predictors of low coverage and selecting pro-equity strategies to address disparities, particularly in the context of COVID-19-related disruptions to immunization that have disproportionately affected poorer and more vulnerable populations.^71^ The behavioral and social drivers of vaccination tools^66^ help to fill this gap, as they collect data on gender and other demographic data to help explain what drives vaccine uptake. Further work to address this gap is needed, as is the need to understand the role of gender, diversity, and inclusion in strengthening decision-making about immunization.

The absence of indicators in our study on disability, diversity, and inclusion was a gap that requires further work to address.

Although 11 of the 17 indicators examining integration of immunization services related to co-delivery of immunization alongside other health services (e.g., antenatal care) or in nontraditional settings (e.g., schools), it is unclear if this is the best way to measure integration of primary care services.^72^ True integration to achieve universal health coverage requires integration across the system in planning, financial resourcing, training and supervision, and community engagement, and current indicators to measure integration do not reflect this.^73^ The use of an integration index has been proposed as a way forward^72^ but runs the risk of measuring concurrent performance across programs without actually measuring the extent to which programs are integrated. Future efforts to define indicators should also consider quantifying the health and efficiency gains that are expected to occur following integration.

The M&E resources in this review did not identify any indicators examining the capacity or competency of the public health workforce or the surge clinical capacity available to draw on in the event of a public health emergency. The need for defining, mapping, and measuring the workforce, including the public health workforce, is recommended in WHO’s Global Strategy on Human Resources for Health 2030.^74^ A framework of immunization workforce competencies, such as WHO’s roadmap for public health workforce developed by Traicoff et al.,^25^ can help countries to establish what the minimum skills and competencies needed are and track suitable indicators and numbers of staff trained in those technical competencies. The Joint External Evaluation Tool, which aims to support countries to develop their capacity to prevent public health threats, provides additional metrics and targets for the public health workforce and surge capacity required, particularly in the context of a public health emergency.^75^ Incorporating these core competencies into public health and clinical training programs can secure surge capacity in the event of future acute emergencies and increase the skills mix of primary health care providers, contributing toward the goal of universal health coverage.

Our study identified 47 distinct indicators across 9/20 (45.0%) M&E resources that measure the impact of immunization programs on disease burden and elimination targets. This small number is likely due to (1) our inclusion criteria and focus on metrics assessing immunization system performance and (2) exclusion of resources that did not measure the operational aspects of immunization systems. Although the indicators identified disease-specific and some summary metrics of disease burden and disease elimination and eradication targets, metrics evaluating wider economic and societal benefits, such as reductions in poverty, improvements in productivity, and financial returns on investment,^76^ were absent. Including these metrics in evaluations of immunization system performance can help to build the case for financial investment in immunization systems and assist decision-making through comparisons with other initiatives or packages of interventions. However, the societal and financial impacts of immunization have often been estimated through modeling studies,^77^^,^^78^ and it may be challenging for resource-restricted countries to conduct these analyses in the short term.

### Strengths and Limitations

To our knowledge, this is the first published review of indicators used to measure system-wide performance of national immunization systems that includes M&E resources published over 2 decades. This review intentionally focuses on M&E resources used globally. Previous global immunization strategies were viewed as top-down strategies, but there is growing recognition that country-specific goals and targets are needed.^4^ Our review collates the indicators used to measure immunization system performance and provides a starting point for national governments and partner agencies to consider how best to assess performance in their own contexts. Additionally, it draws attention to gaps in indicators used to date, highlighting where development partners and researchers can focus their efforts to develop and validate indicators.

Our review provides a starting point for national governments and partner agencies to assess immunization system performance in their own contexts.

Importantly, our study highlights the large burden of data collection and routine reporting for immunization alone, with 169 indicators identified in the 2019 WHO-UNICEF Joint Reporting Form and 112 indicators for national monitoring in the IA2030 M&E framework. We removed 93 duplicate indicators identified in our review but were still left with 631 indicators. Many indicators measured the same construct but in slightly different ways, which means that data collected with 1 resource may not be appropriate for use with another resource, resulting in duplication of efforts if reporting on those indicators is required by development or financial partners. The workload associated with collecting and reporting data using different M&E resources is significant, especially in resource-constrained settings, where dedicating resources to meet reporting requirements has opportunity costs.^12^^,^^79^^,^^80^ Data collection, analysis, and reporting require dedicated financing, resourcing, and accountability mechanisms, potentially diverting resources from other areas of the health system. Countries with constrained systems likely have a greater proportion of their immunization system costs covered by donors and international partners and thus have greater data reporting responsibilities despite having fewer resources to do this. Previous M&E resources have also taken a top-down approach that does not account for variability in country contexts, resulting in reduced country commitment to achieving global immunization goals.^4^ Further research and coordination on which indicators are most important to measure uniformly across all countries is needed, with clear justification of how these indicators are linked to public health benefits to justify resourcing to collect and report this data. For the remainder, it may be more suitable to identify and focus on key indicators based on the values and challenges of the local context, which aligns with the IA2030 strategy’s call for countries to select indicators based on their needs.^13^

Although we systematically searched the literature, we identified only 6 peer-reviewed publications. This is unsurprising as the majority of widely used M&E resources are published in the gray literature by global partner agencies. The Medical Subject Headings used in the MEDLINE database for this topic area are not well-defined and provide a substantial volume of false results. A further limitation is that we only included resources that were publicly available, whereas many countries may report on performance directly to their funders. We ensured that our search strategy captured the Medical Subject Headings against which the identified articles were indexed, and 2 authors of this article are content area experts who checked that we included all major or influential M&E resources. We excluded some widely used and well-established resources, particularly those published by the WHO’s Immunization Analysis and Insights group, such as the Effective Vaccine Management assessment tool and vaccine-specific post-campaign assessment resources (e.g., for COVID-19 and influenza vaccines). Some of these were focused on a specific aspect of immunization systems rather than system-wide performance or had a disease-specific or context-specific focus. M&E resources on child health programs, such as MEASURE Evaluation’s *A Guide for Monitoring and Evaluating Child Health Programs*,^81^ were not included due to their focus on child health more broadly, of which immunization is a smaller component, and on measuring outcomes of the system rather than the performance of its components. Nevertheless, these are important assessment resources whose use can contribute toward strengthening immunization system components.

We did not conduct a quality assessment of indicators using a standardized quality assessment framework. Not all of the indicators were clearly defined, and few were accompanied by a data dictionary clarifying how to calculate statistics and where to source data. It is possible that some indicators may be interpreted differently depending on the context. Although most resources were developed through iterative and consultative processes, it is unclear if indicators have been validated and are associated with improvements in performance. Thus, the existence of M&E resources and indicators does not necessarily mean they are used or provide insightful information. It is likely that countries are tailoring assessments to suit their contexts and measuring indicators that best match their goals and available resources. Furthermore, performance indicators often have limited use in understanding the drivers of performance. Several M&E resources provide guidance on using other data collection methods, such as interviews and focus groups, to generate information to supplement quantitative indicators that together can inform future actions. Further research is needed to better understand how these evaluation resources are used, whether they drive improvements in performance and the pathways for doing so, and which system-level indicators most closely correlate with improvements in vaccine coverage, equity, and reductions in disease. Measuring implementation strength (i.e., the amount of input or activity to support program implementation),^82^ a construct that did not appear in the resources included in our review, may be another way to cumulatively interpret data from selected indicators but requires research to determine which group of indicators can do so most effectively. Developers of M&E resources should assess the quality of indicators included to ensure they are measurable, easy to interpret by users, linked to performance improvements, and relevant to decision-making.

Developers of M&E resources should assess the quality of indicators included to ensure they are measurable, easy to interpret, linked to performance improvements, and relevant to decision-making.

We elected to use the WHO health system framework as the basis for the components of immunization systems, which we used to categorize indicators. This framework has rightly been criticized for focusing on the infrastructural and resource-related components of health systems and insufficiently accounting for the interdependencies between components as well as intangible components such as trust and social value.^83^ In our analysis of indicators, we found that several indicators could have been classified under more than 1 system component, reflecting the interdependencies and connectedness of the components underlying the immunization system. The indicators we identified did not explicitly examine the interactions between components. As discussed earlier, the quality of the indicators was not assessed, and it is unknown if performance of 1 component is linked to or predictive of performance of another. Nevertheless, this framework provides a useful basis to describe the components that describe the health system and its basic functions and has been used to examine the impact of various initiatives on the immunization and health system.^22^^,^^23^

Finally, the inductive method we used to identify measurement areas based on the themes that emerged from our review introduced unavoidable risk of measurement and selection bias. As content area experts, we acknowledge that our prior experiences, assumptions, and beliefs have the potential to influence the research process. We have attempted to minimize this bias by having 2 authors independently code indicators and agree on the definitions set out in Supplement 1.

## CONCLUSIONS

This review identified a multitude of indicators to measure immunization system performance. We identified heterogeneity in metrics assessing the performance of some immunization system components; further studies are needed to reach consensus on how to measure performance in these areas. This summary of indicators can inform country-specific approaches to measuring system performance moving forward, particularly as countries seek to evaluate the impact of the COVID-19 pandemic and leverage the investments made during the response. As countries look to identify focus areas for improvement, they can benefit from selecting indicators that are aligned with national goals, values, and priorities and establishing accountability frameworks to monitor performance and take action. Establishing a country-focused set of core indicators and improving the quality of data on these select metrics will position decision-makers to have better access to data that is useful in decision-making, enabling countries to achieve their immunization goals.

## Supplementary Material

GHSP-D-22-00555-supplement.pdf

## Notes

**Peer Reviewed**

**First published online:** June 7, 2023.
