# Supplementary material for: Measuring National Immunization System Performance: A Systematic Assessment of Available Resources
Source: Glob Health Sci Pract. 2023 Jun 21;11(3):e220055. doi: 10.9745/GHSP-D-22-00555 (PMC10285727; doi:10.9745/GHSP-D-22-00555)
Supplement: GHSP-D-22-00555-supplement.pdf [file GHSP-D-22-00555-supplement.pdf]

Supplement to: Patel C, Rendell N, Sargent GM, et al. Measuring national immunization system performance: a systematic assessment of available resources. *Glob Health Sci Pract*. 2023;11(3):e220055.
https://doi.org/10.9745/GHSP-D-22-00555

Measuring the performance of immunization systems: a literature review

Table of Contents

Supplement 1: Classification framework and definitions used to categorize indicators 2

Supplement 2: Search strategy 8

Supplement 3: Immunization system impacts, outcomes and system components covered by the 19 monitoring and evaluation tools included in this review 10

References 14

Supplement to: Patel C, Rendell N, Sargent GM, et al. Measuring national immunization system performance: a systematic assessment of available resources. *Glob Health Sci Pract*. 2023;11(3):e220055. https://doi.org/10.9745/GHSP-D-22-00555

Supplement 1: Classification framework and definitions used to categorize indicators

Framework

- System impact
  - Disease burden due to vaccine-preventable diseases (VPDs)
  - Achievement of elimination or eradication target
  - Occurrence of outbreaks due to VPDs
  - Summary metrics of disease burden
- System outcomes
  - Vaccination coverage (individual vaccines or multiple vaccines)
  - Equity of vaccination coverage
  - Dropout of vaccination coverage
  - New vaccine introduction
- Demand generation
  - Community engagement
  - Vaccine demand, knowledge, and confidence
- Financing
  - Costs of vaccines and programs
  - Financial planning
  - Government spending on immunization
  - Total expenditure on immunization from all sources
- Governance, program planning and management
  - Existence of policies, processes, and plans
  - Program management
  - Program coordination
  - Plan or process for monitoring and evaluation
- Information systems
  - Data quality
  - Use of data
  - Immunization data systems and processes
  - Vaccine preventable disease surveillance
- Regulation and pharmacovigilance
  - Safety surveillance
  - Regulatory policies and processes
- Service provision
  - Integration of immunization with other health services
  - Activities to reach disadvantaged or under-immunized populations
  - Provision of immunization services
- Vaccines logistics, products, and supplies
  - Availability of vaccines and supplies
  - Effective management of vaccines
  - Use of innovation
- Workforce
  - Training and supervision of health workers
  - Availability or quantity of health workers
  - Health worker competence
  - Working conditions

Definitions

Immunization system: all the components of the health system necessary to deliver vaccines, including all organizations, institutions, resources, processes and activities involved in the provision of immunization programs.

System impact: Indicators describing the overall impacts and goals of immunization systems. Includes reductions in disease burden metrics, achievement of disease eradication or elimination goals, and summary metrics of disease burden like all-cause mortality.

> Disease burden due to VPDs: Encompasses metrics related to measuring the disease burden, such as incidence of a specific disease or deaths or disability-adjusted life-years attributable to a specific VPD.
>
> Achievement of elimination or eradication target: Encompasses metrics related to elimination or eradication goals, such as certification of rubella elimination or achievement of disease incidence of AFP below eradication threshold.
>
> Occurrence of outbreaks due to VPDs: Related to incidence of outbreaks attributable to VPDs.
>
> Summary metrics of disease burden: disease burden metrics that are not specific to VPDs e.g., under-5 mortality rate, all-cause mortality rate.

System outcomes: Indicators describing the outcomes of immunization systems, i.e., goals that are achieved when all components of the system are functioning well and coordinated. Largely related to coverage, expanding breadth of vaccines in national programs, and successful and sustained introduction of new vaccines.

> Vaccination coverage: Indicators related to coverage of vaccines. Includes indicators for coverage of individual specific vaccines, coverage of multiple vaccines (e.g., full vaccination coverage rate including complete coverage of MCV, DTP, BCG, IPV/OPV, etc.), coverage in under-immunized or specific at-risk populations, and zero-dose coverage (i.e., those who have never been vaccinated).
>
> Equity of vaccination coverage: Indicators related to equity of coverage by various factors like geographical (e.g., equity of coverage across districts), gender, education, poverty, and others.
>
> Dropout of vaccination coverage: Indicators related to dropout of coverage, i.e., incomplete coverage of a course of vaccination. Usually related to DTP vaccine or MCV.
>
> New vaccine introduction: Indicators related to the introduction or addition of new vaccines into national immunization programs, implementation of new programs, and sustainability of newly introduced vaccines. Indicators about a specific aspect/system component of new vaccine introduction (e.g., method of funding new vaccine) are under different categories.

Demand generation: Demand generation includes building demand for vaccines in communities and is related to community sentiment about vaccines and vaccine confidence. It also includes community mobilization i.e., activities to engage with communities to educate them about vaccines, and build trust, confidence and acceptance of vaccines.

Community engagement: Indicators related to activities, strategies and/or plans to engage with communities and promote immunizations. Includes metrics related to financial commitment and planning for community engagement.

Vaccine demand, knowledge, and confidence: Indicators related to the demand for vaccines among populations. Includes indicators related to evaluating the value of vaccines, knowledge about vaccines among the public, and support for vaccines by the public and other actors.

Financing: encompasses how immunization programs are funded by countries (both government expenditure and total from all sources), including planning for funding.

Costs of vaccines and programs: Indicators related to the costs of vaccines.

Financial planning: Indicators related to planning for financing or budgeting for vaccines and programs, including activities related to financial planning and whether the funds budgeted are adequate.

Government spending on immunization: Indicators related to government spending on immunization systems, including those examining government spending on immunization systems relative to other sources.

Total expenditure from all sources: Expenditure on immunization systems (vaccines and programs) from all sources including government, donor, and other sources.

Governance, program planning, and management: Governance of immunization systems encompasses the agencies responsible for leading and overseeing immunization programs and the processes by which decisions regarding immunization programs are made. It encompasses metrics about NITAGs (who typically guide national immunization policies and governments' decision-making about vaccination programs). Program planning and management encompasses activities pertaining to the planning and management of immunization programs, including developing microplans and country multi-year plans.

Existence of policies, processes and plans: Indicators examining the existence of specific policies, processes and plans for immunization, such as processes for activating VPD outbreak response, existence of annual plan or microplans in districts, updating of plans, and existence of policies related to specific aspects of immunization program (e.g., injection safety).

Program management: Indicators examining how immunization systems are managed and governed. This includes indicators related to the existence and processes of NITAGs.

Program coordination: Indicators examining coordination of immunization systems/programs, including communication between different levels of government and/or health systems.

Plan or process for monitoring and evaluation: Indicators examining the existence and processes for monitoring and evaluation and provision of feedback, and mechanisms for accountability.

Information systems: all systems and processes that collect, analyze and report data on immunization particularly on immunization coverage and VPDs (note: safety is covered under pharmacovigilance), including how data is used to inform planning and decision making on immunization systems and programs.

Data quality: Indicators examining the quality of immunization data, including timeliness, accuracy, consistency, precision, reliability, and validity.

Use of data: Indicators examining whether and/or how data is used to inform immunization program planning and decision-making.

Immunization data systems and processes: Indicators on the availability, existence and use of specific systems and processes in place to collect, analyze, report and access immunization data, e.g., whether a specific coverage survey was carried out in the previous year, or what proportion of the population have access to their immunization records, or use of specific digital tools like electronic immunization registers or electronic logistics management systems.

Vaccine preventable disease surveillance: Indicators describing VPD surveillance capabilities and methods for VPD surveillance in the country. Related to the ability to identify and detect an outbreak, particularly laboratory capacity.

Regulation and pharmacovigilance: Regulation encompasses assessing vaccines and authorizing their use according to certain criteria pertaining to their quality, safety, and effectiveness. Pharmacovigilance involves monitoring the safety of vaccines used in a country including detecting, assessing, understanding, and preventing adverse effects following immunization.

Safety surveillance: Indicators related to surveillance of vaccine safety, including methods used for vaccine surveillance and adequacy of surveillance (e.g., indicators measuring the numbers of AEFIs).

Regulatory policies and processes: policies and processes to assess vaccines and authorize their use. Includes quality assurance of vaccines.

Service provision: the safe provision of vaccination services. Includes provision of fixed services at health facilities and outreach services.

Integration of immunization with other health services: Indicators measuring the degree of integration of immunization services with other health services like vitamin A supplementation, other primary health care services.

Activities to reach disadvantaged or under-immunized populations: Indicators measuring service provision specifically to under-immunized or never-reached populations, or activities undertaken to deliver services to these populations.

Provision of immunization services: Indicators measuring service provision in any setting (fixed, outreach, supplementary immunization activities). Includes indicators on the number of doses of a vaccine administered, immunization sessions conducted.

Vaccines logistics, products, and supplies: includes the vaccines themselves as well as the supplies required to safely transport them to the site where they are used, store and administer them and dispose of any waste. Encompasses management of vaccines, including cold chain and supply chain management.

Availability of vaccines and supplies: Indicators examining the availability of vaccines at the point of service. This is not about new vaccine introduction or availability of all vaccines recommended by WHO, but availability of vaccines currently recommended by the country. Includes indicators related to stockouts of vaccines, either at the national or district level or point of service (health facilities).

Effective management of vaccines: Indicators related to vaccine and supply chain management, including management of cold chain and wastage. Includes management of vaccines from storage to point of vaccine administration, including transport of vaccines.

Use of innovation: Indicators related to activities undertaken to increase the types of vaccines or innovative ways of delivering vaccine. Includes research activities undertaken related to new vaccines.

Workforce: all workers involved in any function within the immunization system. This includes healthcare providers and skilled workers to manage vaccine supply chains and logistics, monitor and evaluate programs, plan programs and formulate immunization policy using evidence, conduct disease surveillance, mobilize and communicate with communities, monitor vaccine safety and investigate and respond to outbreaks.

Training and supervision of health workers: Indicators related to provision or receipt of supervision of health workers involved in vaccination or training opportunities or other opportunities for capability development and quality improvement for health workers.

Availability or quantity of health workers: Indicators measuring the quantity of health workers (e.g., density of health workers per 100,000 population) or availability of health workers in a given setting, e.g., each health facility has at least one staff member trained in vaccination.

Health worker competence: Indicators measuring the level of staff knowledge and ability to perform tasks correctly, e.g., in relation to their knowledge about vaccines, correct

administration procedures, communication with patients (e.g., provided patient with information on when to return for next dose), correct safety/waste disposal procedures.

Working conditions: Indicators related to working conditions of staff involved in immunization.

Abbreviations
AEFI: adverse event following immunization
AFP: acute flaccid paralysis
BCG: Bacillus Calmette–Guérin (vaccine)
DTP: diphtheria-tetanus-pertussis (vaccines
IPV: inactivated polio vaccine
MCV: measles-containing vaccine
OPV: oral polio vaccine
NITAG: national immunization technical advisory group
VPD: vaccine-preventable disease
WHO: World Health Organization

Supplement to: Patel C, Rendell N, Sargent GM, et al. Measuring national immunization system performance: a systematic assessment of available resources. *Glob Health Sci Pract*. 2023;11(3):e220055. https://doi.org/10.9745/GHSP-D-22-00555

Supplement 2: Search strategy

We searched for monitoring and evaluation tools for immunization systems in the peer-reviewed (OVID Medline) and grey literature (Google Scholar). In Medline, we searched using Medical Subject Heading (MeSH) terms and free-text. Key terms were related to immunization, immunization systems, evaluation, indicators, measurement and surveillance. We used adjacency operators to increase specificity of search terms related to immunization systems, to reduce false hits unrelated to immunization systems, such as titles relating to immune systems or vaccine drug delivery systems. We used truncation to ensure variant endings of terms were included. Articles were also identified by snow-balling through references of full-text included publications. We used Google Scholar to search the grey literature as it captures literature from a broad range of sources. We reviewed the first 1,000 results (ordered by relevance) as the likelihood of identifying further relevant publications beyond the first 1,000 was considered to be low.[1] Searches of the grey literature were limited to evaluation tools published by global implementing partners and did not include international development funding partners who were not directly involved in implementing programs due to the differing priorities and motivations of these organizations. Documents from peak organizations involved in implementing immunization programs globally were reviewed, including the World Health Organization (WHO), the Global Vaccine Alliance (Gavi) and the United States Agency for International Development (USAID). As we sought to identify monitoring and evaluation tools that were commonly used on a global scale, we did not search the government websites of individual countries. Searches were conducted between March (Medline) and May (Google scholar) 2022.

Medline

1 exp *Immunization/ (86009)
2 exp *Immunization Programs/ (9652)
3 exp *Vaccines/ (194241)
4 1 or 2 or 3 (244368)
5 exp Epidemiological Monitoring/ (8460)
6 exp Registries/ (111478)
7 exp Health Information Systems/ (1511)
8 exp Population Surveillance/ (73846)
9 exp Public Health Surveillance/ (5130)
10 exp Data Systems/ (209)
11 ((immuniz$ or immunis$ or vaccin$) adj5 system$).tw. (13006)
12 5 or 6 or 7 or 8 or 9 or 10 or 11 (202749)
13 exp Evaluation Study/ (261647)
14 exp Health Services Research/ (182646)
15 exp "Outcome and Process Assessment, Health Care"/ (1302534)
16 exp Program Evaluation/ (81458)
17 exp Health Impact Assessment/ (891)
18 (dashboard$ or indicator$ or evaluat$ or measur$).tw. (7120965)
19 13 or 14 or 15 or 16 or 17 or 18 (8141009)
20 4 and 12 and 19 (4395)
21 limit 20 to yr="2000 -Current" (3904)

22 limit 21 to English language (3707)

Run on 22 March 2022: 3707 results

Google Scholar

("immunization system" OR "immunization system”) (indicator OR dashboard OR evaluate OR measure OR assess)

Run on 9 May 2022: 3520 results (first 1,000 reviewed)

Supplement to: Patel C, Rendell N, Sargent GM, et al. Measuring national immunization system performance: a systematic assessment of available resources. *Glob Health Sci Pract*. 2023;11(3):e220055. https://doi.org/10.9745/GHSP-D-22-00555

Supplement 3: Immunization system impacts, outcomes and system components covered by the included monitoring and evaluation resources

Author	Name of tool	Year published*	Impact	Outcome	Immunization system components							
					Demand generation	Financing	Governance, program planning, & management	Information systems	Regulation & pharmaco-vigilance	Service provision	Vaccine logistics, products, & supplies	Workforce
WHO[2]	Immunization Agenda 2030 Monitoring and Evaluation Framework	2020	X	X	X	X	X	X	X	X	X	X
WHO & UNICEF[3]	WHO-UNICEF Joint Reporting Form on Immunization (JRF)	2019[#]	X	X	X	X	X	X	X	X	X	X
Cernuschi et al[4]	Gavi indicators for sustainable immunization systems	2018			X	X	X	X	X	X		X
Gavi[5]	Gavi 2016-2020 Strategy Indicators	2018	X	X	X	X	X	X	X		X	
WHO[6]	Reaching Every District Monitoring Tool	2018		X	X	X	X		X		X	X
WHO[7]	A guide for conducting an Expanded Programme on Immunization (EPI) Review	2017	X	X	X	X	X	X	X	X	X	X

Supplement to: Patel C, Rendell N, Sargent GM, et al. Measuring national immunization system performance: a systematic assessment of available resources. *Glob Health Sci Pract*. 2023;11(3):e220055. https://doi.org/10.9745/GHSP-D-22-00555

**Author**	**Name of tool**	**Year published***	**Impact**	**Outcome**	**Immunization system components**							
					**Demand generation**	**Financing**	**Governance, program planning, & management**	**Information systems**	**Regulation & pharmaco-vigilance**	**Service provision**	**Vaccine logistics, products, & supplies**	**Workforce**
National Vaccine Advisory Committee (USA)[8]	Proposed indicators to advance vaccine and immunization efforts in the United States	2017	X	X	X	X	X	X	X	X	X	
Poy et al[9]	Indicators for Immunization Systems Management Group Routine Immunization Dashboard	2017		X			X	X	X		X	X
USAID[10]	USAID MCSP Indicators that describe the strength of the routine immunization system	2017			X		X	X	X		X	X
USAID [11]	USAID Monitoring and Evaluation of the Reaching Every Child-Quality Improvement (REC-QI) approach	2016			X	X	X	X	X		X	X
Tegegne et al[12]	Accountability Framework for the Nigeria Polio Program	2016		X			X	X	X		X	X

**Author**	**Name of tool**	**Year published***	**Impact**	**Outcome**	**Immunization system components**							
					**Demand generation**	**Financing**	**Governance, program planning, & management**	**Information systems**	**Regulation & pharmaco-vigilance**	**Service provision**	**Vaccine logistics, products, & supplies**	**Workforce**
Sodha & Dietz[13]	Indicators that can be used to monitor immunization performance	2015		X			X	X	X		X	X
Shuaib et al[14]	Accountability Framework for Routine Immunization, Nigeria	2014		X		X		X	X		X	X
WHO[15]	Global Vaccine Action Plan: Monitoring and Evaluation/ Accountability Framework	2013	X	X	X	X	X	X	X			X
WHO[16]	New Vaccine Post-Introduction Evaluation (PIE) Tool	2010		X			X		X	X		X
Griffith et al[17]	Toolkit for assessing the impact of measles eradication activities on immunization services and health systems	2010	X	X		X		X			X	X
Gavi[18]	Gavi & WHO Monitoring National Immunization	2002	X	X	X	X	X	X	X		X	X

Author	Name of tool	Year published*	Impact	Outcome	Immunization system components							
					Demand generation	Financing	Governance, program planning, & management	Information systems	Regulation & pharmaco-vigilance	Service provision	Vaccine logistics, products, & supplies	Workforce
	Systems Using Core Indicators											
WHO[19]	WHO Common Assessment Tool for Immunization Services	2002	X	X	X	X	X	X	X	X		X
WHO[20]	Checklist and indicators for optimizing the impact of polio activities on EPI (draft)	2001		X	X	X	X	X	X		X	X
WHO[21]	WHO Indicators for Monitoring District and National Performance	Unknown		X	X	X	X	X	X	X	X	X

EPI: Expanded Program on Immunization; IA2030: Immunization Agenda 2030; JRF: Joint Reporting Form; LMIC: low- and middle-income country; MSCP: Maternal and Child Survival Program; PIE: post-introduction evaluation; SIA: supplementary immunization activity; REC-QI: Reaching Every Child-Quality Improvement; UNICEF: United Nations Children's Fund; USAID: United States Agency for International Development; WHO: World Health Organization

* Where information was available, the year of publication denotes the year that the indicator tool was published and available for use and is not necessarily the date of publication of the article/report.

# The JRF is revised on a regular basis – this study includes indicators included in the tool in 2019.

Supplement to: Patel C, Rendell N, Sargent GM, et al. Measuring national immunization system performance: a systematic assessment of available resources. *Glob Health Sci Pract*. 2023;11(3):e220055. https://doi.org/10.9745/GHSP-D-22-00555

References

1. Haddaway NR, Collins AM, Coughlin D, Kirk S. The Role of Google Scholar in Evidence Reviews and Its Applicability to Grey Literature Searching. Wray KB, ed. *PLoS ONE*. 2015;10(9):e0138237. doi:10.1371/journal.pone.0138237

2. Immunization Agenda. Immunization Agenda 2030: A global strategy to leave no one behind. Published online 2020. Accessed May 26, 2021. https://www.who.int/teams/immunization-vaccines-and-biologicals/strategies/ia2030

3. World Health Organization, UNICEF. WHO-UNICEF Joint Reporting Form 2019.

4. Cernuschi T, Gaglione S, Bozzani F. Challenges to sustainable immunization systems in Gavi transitioning countries. *Vaccine*. 2018;36(45):6858-6866. doi:10.1016/j.vaccine.2018.06.012

5. GAVI The Vaccine Alliance. *2016-2020 Strategy Indicator Definitions*.; 2018. Accessed February 10, 2021. https://www.gavi.org/sites/default/files/document/gavi-2016-2020-strategy-indicator-definitionspdf.pdf

6. WHO Regional Office for Africa. *Reaching Every District (RED): A Guide to Increasing Coverage and Equity in All Communities in the African Region*. World Health Organization; 2017. Accessed September 30, 2021. https://www.afro.who.int/sites/default/files/2018-02/Feb%202018_Reaching%20Every%20District%20%28RED%29%20English%20F%20web%20v3.pdf

7. World Health Organization. *A Guide for Conducting an Expanded Programme on Immunization (EPI) Review*. World Health Organization; 2017. Accessed April 26, 2023. https://apps.who.int/iris/handle/10665/259960

8. Evaluation of the 2010 National Vaccine Plan Mid-course Review: Recommendations From the National Vaccine Advisory Committee: Approved by the National Vaccine Advisory Committee on February 7, 2017. *Public Health Rep*. 2017;132(4):411-430. doi:10.1177/0033354917714233

9. Poy A, van den Ent MMVX, Sosler S, et al. Monitoring Results in Routine Immunization: Development of Routine Immunization Dashboard in Selected African Countries in the Context of the Polio Eradication Endgame Strategic Plan. *The Journal of Infectious Diseases*. 2017;216(suppl_1):S226-S236. doi:10.1093/infdis/jiw635

10. USAID. Indicators that Describe the Strength of the Routine Immunization System. Published 2018. https://publications.jsi.com/JSIInternet/Inc/Common/_download_pub.cfm?id=21625&lid=3

11. USAID. Strengthening the Routine Immunization System through a Reaching Every Child–Quality Improvement Approach in Uganda: A How-to Guide. Published 2016. Accessed September 30, 2021. https://pdf.usaid.gov/pdf_docs/PA00MC5B.pdf

12. Tegegne SG, MKanda P, Yehualashet YG, et al. Implementation of a Systematic Accountability Framework in 2014 to Improve the Performance of the Nigerian Polio Program. *J Infect Dis*. 2016;213(suppl 3):S96-S100. doi:10.1093/infdis/jiv492

13. Sodha SV, Dietz V. Strengthening routine immunization systems to improve global vaccination coverage. *British Medical Bulletin*. 2015;113(1):5-14. doi:10.1093/bmb/ldv001

14. Shuaib F, Garba AB, Meribole E, et al. Implementing the routine immunisation data module and dashboard of DHIS2 in Nigeria, 2014–2019. *BMJ Glob Health*. 2020;5(7):e002203. doi:10.1136/bmjgh-2019-002203

15. Cherian T, Hwang A, Mantel C, et al. Global Vaccine Action Plan lessons learned III: Monitoring and evaluation/accountability framework. *Vaccine*. 2020;38(33):5379-5383. doi:10.1016/j.vaccine.2020.05.028

16. World Health Organization. *New Vaccine Post-Introduction Evaluation (PIE) Tool*. World Health Organization; 2010. Accessed September 30, 2021. http://apps.who.int/iris/bitstream/handle/10665/70436/WHO_IVB_10.03_eng.pdf;jsessionid=8BF9AA7B5097D9332163FDFB39C05659?sequence=1

17. Griffiths UK, Hanvoravongchai P, Oliveira V, Mounier S, Balabanova D. A Toolkit for Assessing the Impacts of Measles Eradication Activities on Immunization Services and Health Systems at Country Level. :62.

18. Gavi, The Vaccine Alliance. Monitoring national immunization systems using core indicators. Published 2002. Accessed September 30, 2021. https://www.who.int/immunization/monitoring_surveillance/routine/indicators/core_set_international_community.pdf

19. World Health Organization. *The Common Assessment Tool for Immunization Services: Assessment Methodology*. World Health Organization; 2002. Accessed September 29, 2021. https://apps.who.int/iris/bitstream/handle/10665/68871/WHO_IVB_04.05_%28booklet1%29.pdf?sequence=1&isAllowed=y

20. World Health Organization. Checklist and indicators for optimizing the impact of polio activities on EPI, Draft version 1.0 : draft for field testing. Published 2001. Accessed December 6, 2022. https://apps.who.int/iris/handle/10665/66764

21. World Health Organization. Indicators for monitoring district and national performance. Accessed December 6, 2022. https://www.technet-21.org/media/com_resources/trl/873/multi_upload/20xx_Proposed%20Core%20Indicators%20District%20and%20Natioanl%20Level_WHO.pdf
